# Hypoxia-inducible factor-1α overexpression indicates poor clinical outcomes in tongue squamous cell carcinoma

**DOI:** 10.3892/etm.2012.779

**Published:** 2012-10-30

**Authors:** FEI-WU KANG, YIN GAO, LIN QUE, JING SUN, ZUO-LIN WANG

**Affiliations:** 1 Department of Oral and Maxillofacial Surgery, Hospital of Stomatology, Tongji University, Shanghai 200072;; 2 Department of Oral and Maxillofacial Surgery, Hospital of Stomatology, Wenzhou Medical College, Wenzhou 325035, P.R. China

**Keywords:** hypoxia, hypoxia-inducible factor-1α, vascular endothelial growth factor, tongue squamous cell carcinoma, prognosis

## Abstract

The aim of the present study was to investigate the expression of hypoxia-inducible factor-1α (HIF-1α) in tongue squamous cell carcinoma (TSCC) and to assess its possible impact on prognosis. A total of 49 tumor samples and 15 adjacent non-tumor samples from 49 patients treated between January 2000 and December 2005 at the Department of Oral and Maxillofacial Surgery, Hospital of Stomatology, Tongji University (Shanghai, China) were obtained for investigation with immunohistochemistry and reverse transcription-polymerase chain reaction (RT-PCR). The expression of HIF-1α was detected in 87.76% (43/49) of the TSCC samples and in 33.33% (5/15) of the adjacent non-tumor tissues. The expression of vascular endothelial growth factor (VEGF) was also observed in 83.67% (41/49) of the TSCC samples and in only 20% (3/15) of the adjacent non-tumor samples at a low level. RT-PCR revealed that the mRNA expression of HIF-1α and VEGF was present in the tumor tissues; however, it was barely detected in the corresponding adjacent normal tissues. The overexpression of HIF-1α was significantly associated with T classification (P=0.01), lymphatic metastasis (P=0.05) and histological differentiation (P<0.001). Furthermore, HIF-1α overexpression was significantly associated with poor overall (P=0.001) and disease-free survival rates (P=0.01), independent of T stage and lymphatic metastasis. The Cox proportional hazards regression model demonstrated that the level of HIF-1α expression may be an independent prognostic factor for TSCC. HIF-1α overexpression was observed in TSCC and its overexpression suggests a poor prognosis. HIF-1α may be a molecular marker for predicting the prognosis of TSCC.

## Introduction

Hypoxia is a general characteristic of malignant tumors (1,2) which occurs in numerous solid tumors (3–5). It regulates a variety of transcription factors, including hypoxia-inducible factor-1 (HIF-1) which is a heterodimeric transcription factor consisting of 2 subunits; a constitutively stable β subunit and an oxygen sensitive α subunit (6). The α subunit is rapidly degraded through the ubiquitin-proteasome pathway in the physiological microenvironment (7). However, the α subunit is stabilized and accumulates under hypoxic conditions (8) due to the inactivation or absence of the von Hippel-Lindau (VHL) tumor suppressor gene (9,10). HIF-1α products regulate cell adaptation to hypoxic microenvironments by modulating a number of downstream genes involved in vascular growth and cellular metabolism. Among these genes, vascular endothelial growth factor (VEGF) is vital as a regulatory gene of angiogenesis in the adaptation to hypoxic microenvironments (11). Studies have demonstrated the correlation between HIF-1α and VEGF in tumor cells (12) and solid tumors (13) and high levels of HIF-1α expression appear to predict a poor prognosis for various types of cancer (4,14,15).

However, relevant studies on tongue squamous cell carcinoma (TSCC) are rare. As a type of common malignant tumor in the oral cavity, TSCC has a high mortality rate due to early metastasis and recurrence. Although the level of healthcare has greatly improved, the cure rate of TSCC remains unsatisfactory and the 5-year survival rate is ∼50% (16).

The aim of the present study was to investigate the association of HIF-1α expression with various clinical parameters using reverse transcription-polymerase chain reaction (RT-PCR), immunohistochemistry and western blot analysis to evaluate the impact of HIF-1α expression on the prognosis of TSCC.

## Patients and methods

### 

#### Clinical cases

A cohort of 49 patients (28 males and 21 females; mean age, 69.2 years; range, 45–84) was eligible for the present study. All the patients were treated between January 2000 and December 2005 at the Department of Oral and Maxillofacial Surgery, Hospital of Stomatology, Tongji University (Shanghai, China). A total of 49 paraffin-embedded tumor specimens of TSCC and 15 adjacent non-tumor tissue specimens were obtained for immunohistochemistry. Additionally, 15 fresh frozen tumor tissue specimens and their corresponding adjacent tissue specimens were obtained for RT-PCR. All the patients included in the study had a primary tumor in the oral cavity which was diagnosed as a squamous cell carcinoma (SCC) by clinical, radiological and pathological examination. Surgical treatment involved radical resection of the whole tumor with a free histopathological margin of at least 15 mm from the tumor borders. Bilateral selective neck dissection was performed in cases of suspect results from pre-operative tumor staging by computerized tomography and sonographic examination or in cases of a tumor size of >2 cm. The tissues were confirmed post-surgically as TSCC or adjacent non-tumor tissue using hematoxylin and eosin (H&E) staining. All tumors were classified according to the UICC TNM staging system which was revised in 2002 (17).

#### Follow-up

All patients were followed up by telephone or postoperative questionnaires monthly and underwent ultra-sound or computed tomography examinations at least once every 3 months at the outpatient clinic, particularly in the first 2 years. The average overall survival time was 36.73 months (ranging from 3 to 72 months).

#### RT-PCR

Total RNA was isolated from the frozen tissues using the TRIzol reagent (Invitrogen, Carlsbad, CA, USA) and cDNA was synthesized using a PrimeScript RT reagent kit (Takara, Beijing, China). mRNA expression was evaluated quantitatively using real-time RT-PCR with SYBR Premix Ex Taq™ (Takara) and an ABI PRISM^®^ 7900HT real-time PCR system. The thermocycler conditions were pre-denaturing at 95°C for 30 sec, followed by 35 cycles of denaturing at 95°C for 30 sec, annealing at 60°C for 30 sec and extension at 68°C for 1 min. The relative amount of the PCR product was defined as the threshold cycle (CT value) of the sample divided by that of β-actin. All experiments were performed in triplicate. The primers were synthesized (Sangon Biotech, Shanghai, China) and the sequences were as follows: for HIF-1α, the forward primer was 5′-GAA CCT GAT GCT TTA AAC T-3′ and the reverse primer was 5′-CAA CTG ATC GAA GGA ACG-3′; for VEGF, the forward primer was 5′-TTT CTG CTG TCT TGG GTG CAT TGG-3′ and the reverse primer was 5′-TCT GCA TGG TGA TGT TGG ACT CCT-3′; for β-actin, the forward primer was 5′- TGG, CAC, CCA, GCA, CAA, TGA, A- 3′ and the reverse primer was 5′-CTA AGT CAT AGT CCG CCT AGA AGC A-3′.

#### Immunohistochemisty

Sections (thickness, 4 *μ*m) of paraffin-embedded TSCC and adjacent tissues were dewaxed in xylene and rehydrated. Antigen retrieval was performed by heating the sections with 10 mM citrate buffer (pH 6.0) for 15 min in a microwave. The sections were washed in PBS buffer and blocked for 30 min in 10% goat serum containing 1% BSA and 0.02% Triton X-100. Serial sections were incubated with rabbit anti-human HIF-1α 67 monoclonal antibody (1:100; Santa Cruz Biotechnology Inc., Santa Cruz, CA, USA) and rabbit anti-human VEGF polyclonal antibody (1:100; Santa Cruz Biotechnology Inc.) separately for 20 min (with PBS instead of primary antibody as the negative control) and washed in PBS 3 times for 15 min. Subsequently, a catalyzed signal amplification system (Santa Cruz Biotechnology Inc.) was used for HIF-1α staining according to the manufacturer’s instructions. The antibodies were detected using a standard avidin-biotin complex method [biotinylated rabbit anti-mouse antibody (Santa Cruz Biotechnology Inc.) and an avidin-biotin complex (Santa Cruz Biotechnology Inc.)] and developed with diaminobenzidine. All sections were then counterstained for 45 sec with hematoxylin and dehydrated in alcohol and xylene prior to mounting. Non-tumor tissue samples adjacent to the paraffin-embedded tumor specimens were examined in a similar way for H&E HIF-1α staining.

#### Classification of HIF-1α expression

The HIF-1α protein was mainly present inside the nucleus, shown as brown or brown-yellow granules located inside the tumor cell. The VEGF protein was present inside the tumor cell or the cell membrane. Each section was examined independently by 2 pathologists. Five fields of view were randomly selected under an optical microscope at a x200 magnification. The positive cells were examined for staining intensity and counted 3 times to calculate the average number in each section. Based on the relative number of positive cells and staining intensity, 4 levels were defined to identify the staining activity of tumor cells: level I, no positive cells; level II, <10% positive cells, weak staining; level III, 10–50% positive cells, moderate staining; level IV, >50% positive cells, strong staining.

#### Statistical analysis

Correlations between clinicopathological features and the expression of HIF-1α were evaluated using the Chi-square test. Disease-free survival (DFS) and overall survival (OS) were used as the 2 end-points of the survival analysis. OS was defined as the number of months from the day the patient left the hospital to patient mortality. Patients who succumbed to other causes or remained alive at the final follow-up were considered to be review events. DFS was defined as the tumor-free time between the initial treatment and the first local recurrence or distant metastasis. Survival curves of DFS and OS were analyzed using the Kaplan-Meier method. The log-rank test was used to assess the differences between the groups and multivariate survival analysis was performed using Cox’s regression model. P<0.05 was considered to indicate statistically significant differences.

## Results

### 

#### Immunohistochemical analysis of HIF-1α and VEGF expression in TSCC

**Figure 1:**
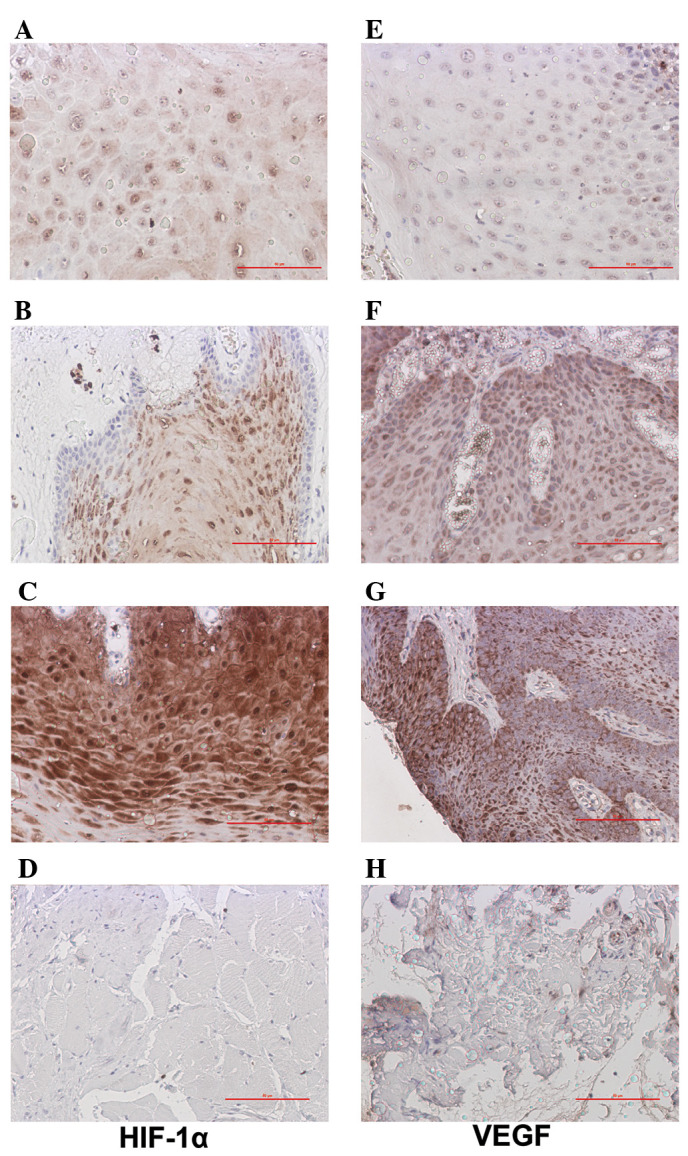
Expression of HIF-1α and VEGF (immunohistochemisty, magnification, x200). (A–C) Expression of HIF-1α at various levels in TSCC: (A) weak positive, (B) medium positive and (C) strong expression. (D) No expression of HIF-1α in the adjacent tissue. (E–G) Expression of VEGF in TSCC. (H) Weak expression of VEGF in adjacent normal tissue. Red lines, 50 *μ*m; HIF-1α, hypoxia-inducible factor-1α; VEGF, vascular endothelial growth factor; TSCC, tongue squamous cell carcinoma.

Fig. 1 shows the results of the immunohistochemisty analysis. In the 49 cases of TSCC, there were 6 (12.24%) negative cases, 21 (42.86%) weak positive cases (Fig. 1A), 12 (24.49%) medium positive cases (Fig. 1B) and 10 (20.41%) strong positive cases (Fig. 1C); the total positive rate was 87.76%. By contrast, there were only 5 (33.33%) cases of weak expression of HIF-1α in the 15 adjacent non-tumor tissue specimens (Fig. 1D). For VEGF, there were 8 (16.33%) negative cases, 17 (34.69%) weak positive cases (Fig. 1E), 16 (32.65%) medium positive cases (Fig. 1F) and 8 (16.33%) strong positive cases in the present study (Fig. 1G) and the overall positive rate was 83.67%. There were only 3 (20%) cases of weak expression of VEGF in the 15 adjacent non-tumor tissue specimens (Fig. 1H). The differences were observed to be significant (P<0.001).

**Figure 2:**
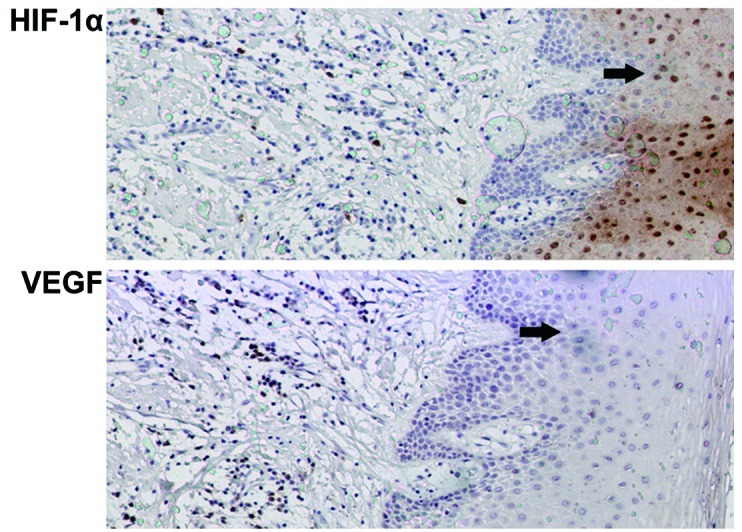
Expression of HIF-1α and VEGF (immunohistochemistry, magnification, x200) in a single section of adjacent non-tumor tissue. HIF-1α and VEGF were expressed at relatively low levels compared with the same regions of the TSCC sections. Arrows indicate cell nuclei at the same region of the serial TSCC sections. HIF-1α, hypoxia-inducible factor-1α; VEGF, vascular endothelial growth factor; TSCC, tongue squamous cell carcinoma.

HIF-1α was observed to be markedly expressed in the TSCC tissue, while no expression was observed in the adjacent tissue of the representative section shown in Fig. 2. In the same regions, HIF-1α and VEGF were expressed at relatively low levels. This was supported by the results of the RT-PCR.

#### RT-PCR analysis of HIF-1α and VEGF expression in TSCC

**Figure 3:**
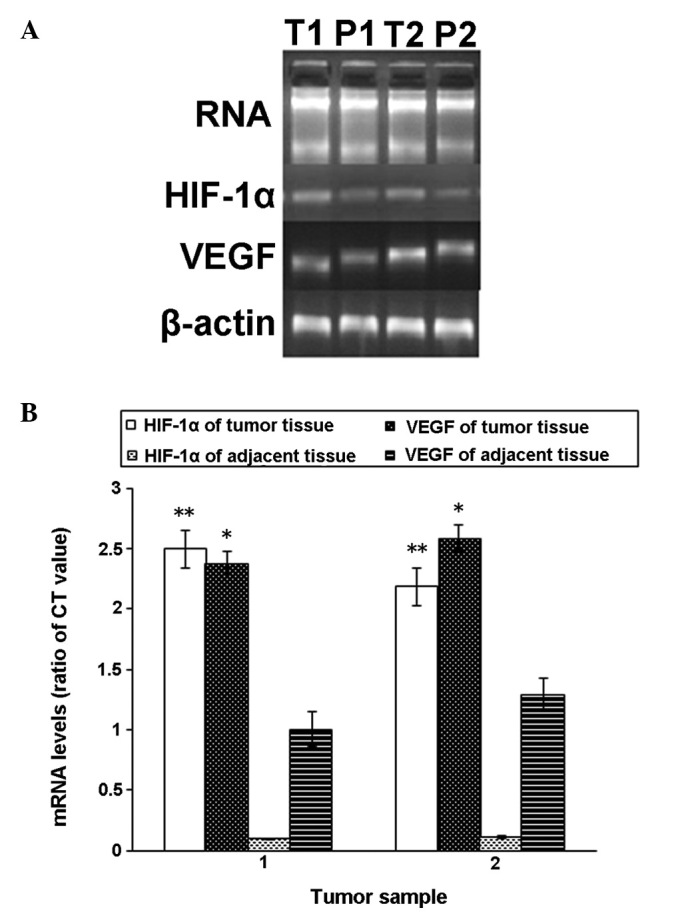
Expression levels of HIF-1α and VEGF in fresh tissue samples. (A) mRNA expression levels of HIF-1α and VEGF by RT-PCR between (T1, T2) tumor and (A1, A2) adjacent tumor samples (2 cases). (B) Quantitative analysis of HIF-1α and VEGF mRNA expression levels (^*^P<0.05, ^**^P<0.001, compared with adjacent tissue). HIF-1α, hypoxia-inducible factor-1α; VEGF, vascular endothelial growth factor; RT-PCR, reverse transcription-polymerase chain reaction.

As shown in Fig. 3A, the mRNA expression levels in fresh tissues obtained from 2 cases were observed, demonstrating that HIF-1α mRNA was highly expressed in the tumor tissue but expressed at a low level in the adjacent tissue. However, VEGF mRNA expression was observed in the tumor tissue and the adjacent tissue, although the expression in tumor was higher. These data indicated that HIF-1α expression was markedly higher in tumor tissue when compared with that of the corresponding adjacent tissue specimens and that for VEGF, the difference in mRNA expression levels was less significant (Fig. 3B).

#### Association between HIF-1α and clinicopathological parameters

Table I shows the correlation between the parameters, including gender, age, histological differentiation, surgical approach, lymphatic metastasis, T stage and HIF-1α expression. In the present study, the 4 classes of expression of HIF-1α were combined into 2 classes: class I/II and class III/IV. No significant correlation was observed between gender, age, surgical approach and expression of HIF-1α (class I/II and class III/IV) using the Chi-square test. However, the T stage (Tis/T1 vs. T2/T3) and histological differentiation (G1 vs. G2 + G3) were observed to correlate with HIF-1α overexpression and the results were statistically significant (P<0.05).

### Association between HIF-1α and prognosis

#### Univariate analysis of clinical parameters with DFS and OS

Table II shows the results of the Kaplan-Meier analysis. The results revealed significantly poorer DFS (P<0.05) and OS (P<0.05) with histological differentiation (P= 0.020, P=0.008), lymphatic metastasis (P<0.001, P<0.001) and HIF-1α expression (P= 0.001, P<0.001). No significant associations between survival rates and gender, age or neck dissection were observed.

#### Multivariate analysis of clinical parameters with DFS and OS

In multivariate Cox analysis the OS and DFS were compared according to clinical parameters (lymphatic metastasis, histological differentiation) with the HIF-1α expression. Lymphatic metastasis and HIF-1α expression were identified by Cox regression as independent predictors of DFS and OS. Lymphatic metastasis (P=0.010, P=0.011) and HIF-1α expression (P=0.050, P=0.030) were also predictors of tumor-free survival in multivariate regression (Table III).

**Figure 4:**
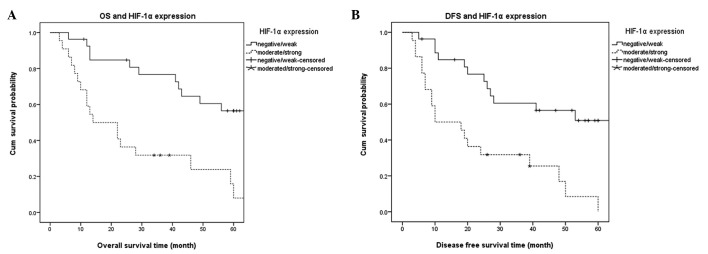
Survival analysis of HIF-1α overexpression with overall survival (OS) and disease-free survival (DFS) (Kaplan-Meier curve). The survival timetable of all patients was divided into two groups for comparison based on the expression levels of HIF-1α: Negative/weak group and Moderate/strong group. (A) OS curve; (B) DFS curve. HIF-1α, hypoxia-inducible factor-1α.

Fig. 4 shows the Kaplan-Meier survival curve analysis of DFS and OS. The expression of HIF-1α (class I/II and class III/IV) affected the OS (Fig. 4A) and DFS (Fig. 4B) of patients with TSCC. The patients with high HIF-1α expression levels had poorer DFS and OS, suggesting that the overexpression of HIF-1α was associated with a poor prognosis.

## Discussion

HIF-1α is the most significant nuclear transcription factor identified as mediating the hypoxic response. As a global regulatory factor, it is able to activate a wide range of genes mediating physiological responses to hypoxia and consequently regulates oxygen concentration in cell metabolism (18). The transcriptional activity of HIF-1α is activated by hypoxia, thus triggering a series of adaptive responses leading to glucose metabolism, tumor angiogenesis and erythropoietin generation (19). Hypoxia is a general characteristic of malignant tumors which occurs in numerous solid tumors (20). Due to the continuous growth and expansion of the solid tumor, the intratumoral oxygen concentration is continuously reduced until hypoxia occurs. This causes HIF-1α accumulation inside the cell nucleus which activates various types of downstream genes for hypoxia adaptation. As a regulatory gene of angiogenesis in the adaptation to hypoxic microenvironments, VEGF is vital in tumor recurrence and metastasis (21). It is a highly specific vascular endothelial cell mitogen which directly stimulates endothelial cells, thus promoting endothelial cell proliferation, migration and increases in vascular permeability. The normal functions of VEGF include creating new blood vessels during embryonic development and following injury. However, under certain pathological conditions, VEGF may contribute to diseases such as tumors. Studies have demonstrated the function of VEGF in tumorigenesis and revealed it to be an independent indicator for predicting malignant tumors with poor prognoses (22–24). Sugiura *et al* examined 160 oral SCC specimens from the oral cavity using immunohistochemistry and revealed that VEGF-C may be used to predict the lymphatic metastasis of oral SCC (25).

In the present study, the expression of VEGF was observed to be significantly associated with that of HIF-1α. The results of RT-PCR suggested that the HIF-1α was present, overexpressed in TSCC and closely associated with VEGF. These results were consistent with those reported by Yasuda *et al*(13). The authors used *in situ* hybridization and immunohistochemistry to observe the expression of the HIF-1α gene and its association with the VEGF protein and microvessel density (MVD) and revealed that the expression of HIF-1α mRNA positively correlated with the VEGF protein expression and MVD in colorectal adenoma. Additionally, in the present study, HIF-1α and VEGF were mainly expressed in the TSCC tissue and were barely detected in the adjacent non-tumor tissue suggesting that HIF-1α was present and overexpressed in TSCC.

Since a number of studies have revealed HIF-1α overexpression to be significantly associated with poor prognoses in certain solid tumors, including pancreatic cancer, breast cancer, rectal adenocarcinoma and cervical cancer (26–29), it was investigated whether HIF-1α may function as a prognostic factor of TSCC. Using the the immunohistochemical staining performed on the 49 TSCC specimens of HIF-1α, the association between HIF-1α expression and the prognoses of patients with TSCC was studied. The data demonstrated that patients with no or weak expression of HIF-1α had higher survival rates (approximately 60%) than those with moderate or high expression of HIF-1α (approximately 30%). HIF-1α overexpression was closely associated with clinicopathological parameters, including histological differentiation, T stage and lymph node metastasis. The data also revealed unfavorable effects on survival rate. In multivariate Cox regression analysis, lymphatic metastasis and HIF-1α expression were significantly associated with DFS and OS, suggesting that the subgroup of patients with HIF-1α overexpression may have a high risk of TSCC and a poor prognosis. This hypothesis was supported by a number of studies in various research fields as mentioned previously. Bos *et al*(30) investigated the expression levels of HIF-1α, HER-2/neu, estrogen receptor and progesterone receptor in 150 patients with early-stage breast carcinoma using immunohistochemistry and HER-2/neu gene amplification with automated fluorescent *in situ* hybridization. The authors observed that increased levels of HIF-1α were associated independently with lower survival rates in patients with lymph node negative breast carcinoma.

However, the results of certain studies are inconsistent with those of the present study. Fillies *et al*(31) investigated 85 patients with histologically demonstrated surgically treated T1/2 SCC of the oral floor and the results suggested that HIF-1α overexpression was an indicator of favorable prognosis in T1 and T2 SCC of the oral floor. This contradiction may be due to a uniform cut-off point of HIF-1α expression or various types of tendentious treatment for the tumor. Considering this, HIF-1α may function as an independent prognostic marker of TSCC.

In conclusion, the present study is the first to clinically study patients with TSCC in the East China region. The findings suggested that the overexpression of HIF-1α predicts a poor prognosis in TSCC. Further studies should be performed to explore the potential functional role of HIF-1α in malignant tumors and to determine whether HIF-1α may be regarded as an indicator or target in the diagnosis and treatment of TSCC.

## Figures and Tables

**Table I:** Correlation between HIF-1α expression and clinical parameters in TSCC.

		HIF-1α		
Parameter	n (%)	I/II	III/IV	P-value
Total	49 (100)	27	22	-
Gender				0.158
Male	28 (57.14)	18	10	
Female	21 (42.86)	9	12	
Age (years)				0.246
<70	21 (42.86)	14	7	
≥70	28 (57.14)	13	15	
Neck dissection				0.395
Yes	30 (61.22)	15	15	
No	19 (38.78)	12	7	
T stage				0.010^a^
T1 + Tis	24 (48.98)	18	6	
T2 + T3	25 (51.02)	9	16	
Lymphatic metastasis				0.005^a^
Negative	43 (87.76)	27	16	
Positive	6 (12.24)	0	6	
Histological differentiation				0.000^a^
G1	23 (46.94)	21	2	
G2 + G3	26 (53.06)	6	20	

Data were analyzed using the Chi-square test.

a P<0.05 was considered to indicate statistically significant differences. HIF-1α, hypoxia-inducible factor-1α; TSCC, tongue squamous cell carcinoma.

**Table II:** Correlation of clinical parameters with DFS and OS in TSCC.

Parameter	DFS (months)	P-value	OS (months)	P-value
Gender		0.107		0.115
Male	33.00±21.96		39.93±25.04	
Female	26.00±18.99		32.48±21.14	
Age (years)		0.292		0.218
<70	31.81±20.53		39.38±23.60	
≥70	28.64±21.32		34.75±23.675	
Neck dissection		0.094		0.088
Yes	25.53±20.06		31.33±22.561	
No	37.05±20.57		45.26±22.99	
T stage		0.132		0.108
T1 + Tis	31.33±20.31		39.42±22.58	
T2 + T3	28.72±21.66		34.16±24.55	
Lymphatic metastasis		0.000^b^		0.000^b^
Negative	32.98±20.46		40.33±22.78	
Positive	8.67±5.20		11.00±6.29	
Histological differentiation		0.020^a^		0.008^a^
G1	36.35±20.00		46.83±21.96	
G2 + G3	24.38±20.28		27.81±21.45	
HIF-1α expression		0.001^a^		0.000^b^
Negative/weak	37.63±20.61		46.96±21.85	
Moderate/strong	20.64±17.30		24.18±19.30	

Data were analyzed using the log-rank test.

a P<0.05 was considered to indicate statistically significant differences,

b P<0.001. DFS, disease-free survival; OS, overall survival; HIF-1α, hypoxia-inducible factor-1α; TSCC, tongue squamous cell carcinoma.

**Table III:** Multivariate analysis of DFS and OS in TSCC.

	DFS		OS	
Parameter	P-value	RR	P-value	RR
Lymphatic metastasis				
Negative vs. Positive	0.010^a^	0.080–0.705	0.011^a^	0.086–0.732
Histological differentiation				
G1 vs. G2 + G3	0.749	0.329–2.224	0.619	0.296–2.066
HIF-1α expression				
Negative/weak vs. moderate/strong	0.050^a^	0.146–1.001	0.030^a^	0.122–0.898

The Cox partial nonparametric regression model was used to evaluate the predictive power of various combinations of prognosticators in a multivariate manner.

a P<0.05 was considered to indicate statistically significant differences. DFS, disease-free survival; OS, overall survival; RR, relative risk; HIF-1α, hypoxia-inducible factor-1α; TSCC, tongue squamous cell carcinoma.
